# The Role of *ptsH* in Stress Adaptation and Virulence in *Cronobacter sakazakii* BAA-894

**DOI:** 10.3390/foods11172680

**Published:** 2022-09-02

**Authors:** Yi Sun, Jiahui Li, Yanpeng Yang, Gaoji Yang, Yiqi Shi, Shuo Wang, Muxue Wang, Xiaodong Xia

**Affiliations:** 1 College of Food Science and Engineering, Northwest A&F University, Xianyang 712100, China; 2 National Engineering Research Center of Seafood, School of Food Science and Technology, Dalian Polytechnic University, Dalian 116034, China

**Keywords:** *Cronobacter sakazakii*, *ptsH* gene, stress response, RNA-seq

## Abstract

*Cronobacter sakazakii*, an emerging foodborne pathogen that was isolated primarily from powdered infant formula, poses an important issue in food safety due to its high stress tolerance and pathogenicity. The Hpr (encoded by *ptsH* gene) has been shown to regulate carbon metabolism as well as stress response and virulence. However, the functional properties of *ptsH* in *C. sakzakii* have not been investigated. In this study, we clarified the role of *ptsH* in the *C. sakzakii* stress response and virulence, and explored its possible regulatory mechanism by RNA-seq. Compared with wild-type, the Δ*ptsH* mutant showed a slower growth rate in the log phase but no difference in the stationary phase. Moreover, the resistance to heat stress (65 °C, 55 °C), simulated gastric fluid (pH = 2.5), biofilm formation and adhesion to HT-29 cells of Δ*ptsH* mutant were significantly decreased, whereas the oxidative resistance (1, 5, 10 mM H_2_O_2_), osmotic resistance (10%, 15%, 20% NaCl), and superoxide dismutase activity were enhanced. Finally, RNA-seq analysis revealed the sulfur metabolism pathway is significantly upregulated in the Δ*ptsH* mutant, but the bacterial secretion system pathway is dramatically downregulated. The qRT-PCR assay further demonstrated that the Δ*ptsH* mutant has elevated levels of genes that are related to oxidative and osmotic stress (*sodA*, *rpoS*, *cpxA*/*R*, *osmY*). This study provides a great understanding of the role of *ptsH* in diverse stress responses and virulence in *C. sakazakii*, and it contributes to our understanding of the genetic determinant of stress resistance and pathogenicity of this important foodborne pathogen.

## 1. Introduction

*Cronobacter sakazakii* (*C. sakazakii*) is a Gram-negative, motile, and opportunistic pathogen that leads to serious diseases in newborns, including meningitis, bacteremia, or necrotizing enterocolitis, with a death rate of up to 80% [1]. *C. sakazakii* has been isolated from various food products and a wide range of food processing sources [2]. Powdered infant formula (PIF) has been regarded as one of the primary products that is linked to *C. sakazakii* in neonatal infections [3,4]. To establish contamination and infection, *C. sakazakii* has been shown to withstand a variety of challenging environmental stimuli from food chains and the gastrointestinal tract (GIT) [5,6]. For example, *C. sakazakii*’s remarkable resistance to osmotic stress (10% NaCl) and high thermotolerance (50–65 °C) can exacerbate the high residual risk after spray drying and pasteurization of the final product, particularly in PIF processing [5,7]. Furthermore, the strong adaptation of *C. sakazakii* to acid (pH < 3.9) and oxidative (100 mM H_2_O_2_) stress provides a competitive advantage in coping with the acidity in GIF and avoiding the oxidative burst from the innate immune response [5,8].

Biofilm formation is an effective barrier of bacteria to various environmental stressors, whereas attachment to the surface is the first stage in biofilm establishment [9,10]. *C. sakazakii* is capable of forming biofilms on materials that are frequently used in food preparation, such as stainless steel, glass, silicon, and polycarbonate [11]. *C. sakazakii*’s remarkable ability to survive a wide range of extreme stressors, as well as bacterial attachment to various surfaces, poses a serious threat to food safety.

The phosphotransferase system (PTS), which consists of HPr, enzyme I (EI), and enzyme II (EII), primarily controls sugar absorption and carbohydrate metabolism in Gram-negative bacterial species [12]. It has been established that HPr, which is encoded by *ptsH*, is involved in phosphate group transfer, as well as the expression of a number of virulence genes and the development of biofilms [13,14]. Furthermore, HPr is required for meningococcal resistance to oxidative stress [15]. The expression of Hpr was enhanced under acid stress in *Lactobacillus casei*, through a high rate of activity in glucose PTS and ATP generation to maintain a high intracellular pH [16]. So far, the crucial role of carbohydrate metabolism in regulating virulence and stress tolerance in various bacterial pathogens has been widely studied [12,17,18], and numerous studies have dissected the mechanisms underlying *C. sakazakii*’s stress resistance and virulence [19,20,21]. However, the interaction between genes that are involved in carbon metabolism and the stress response or pathogenicity of *C. sakazakii* remains to be clarified.

The purpose of this research was to investigate the functional characteristics of the *ptsH* gene in *C. sakazakii* BAA-894 that was isolated from PIF that was associated with a neonatal intensive care unit outbreak [22]. We constructed a *ptsH* mutant and examined the impact of the *ptsH* gene on cell growth, resistance to heat stress, simulated gastric fluid (SGF), oxidative stress, and osmotic stress. Moreover, the ability of biofilm formation and adhesion to HT-29 cells were detected between the wild-type (WT) and the *ptsH* mutant. Furthermore, RNA-sequencing (RNA-seq) and quantitative real-time PCR (qRT-PCR) were performed to explore the function of *ptsH* on the gene regulation at the genome level. This study contributes to our understanding of the role of *ptsH* in stress adaptation and virulence in *C. sakazakii*.

## 2. Materials and Methods

### 2.1. Bacterial Strains and Plasmids

The bacterial strains and derivative plasmids that were used in this study are listed in Table 1. *Escherichia. coli* S17 *λpir* was used in gene deletion experiments and cultured in LB medium at 37 °C. All the strains were cultured in Luria-Bertani (LB) broth or LB agar at 37 °C with appropriate antibiotics. The antibiotics were added at the following concentrations: 32 μg/mL nalidixic acid (NA) and 20 μg/mL chloramphenicol (Cm).

### 2.2. Construction of the ptsH Mutant

The chromosomal deletion mutants were constructed by *sacB*-based allelic exchange [24]. Two DNA fragments were amplified by PCR with *C. sakazakii* BAA-894 chromosomal DNA as a template with primer pairs UP-F/UP-R and DOWN-F/DOWN-R, respectively (Table 2). Fragment (UP + DOWN) that was amplified with UP-F and DOWN-R was digested with SphI and SalI, and ligated into SphI and SalI digested pDM4, resulting in plasmid pDM4::UP + DOWN. Following sequencing and purification, this recombinant plasmid was transformed into *E. coli* S17 *λpir* by 42 °C heat shock for 90 s, and subsequently transferred into *C. sakazakii* WT strain via conjugation. Single crossover recombination and double crossover recombination events were selected using LB agar plates containing appropriate antibiotics or 10% sucrose, respectively. To confirm the mutant, the primers UP-F/DOWN-R were used.

### 2.3. Growth Analysis of C. sakazakii WT Strain and ΔptsH Mutant

The *C. sakazakii* WT strain and Δ*ptsH* mutant suspensions (approximately 10^5^ CFU/mL) with the appropriate antibiotic were transferred into a 96-well microtiter plate (Nunc, Copenhagen, Denmark) and incubated at 37 °C. A microplate spectrophotometer (Model 680; Bio-Rad, Hercules, CA, USA) was used to measure the optical density at 600 nm (OD_600_) at 2 h intervals.

### 2.4. Environmental Stress Tolerance of C. sakazakii WT Strain and ΔptsH Mutant

Both strains were cultured overnight, centrifuged at 5000× *g* for 5 min at 4 °C, resuspended, and diluted with LB broth to OD_600_ value reached 0.5. The prepared strain samples were diluted 100 times (approximately 10^6^ CFU/mL) and were used in the following experiments.

The heat resistance was investigated according to the previous method [25]. Equilibration of the prepared samples were heated to 65 °C or 55 °C in a temperature-controlled water bath. At 65 °C, the samples were removed at 0, 2, 5, and 10 min. At 55 °C, the samples were taken at 0, 10, 30, 60, 90, and 120 min. All of the samples were quickly cooled in an ice water bath before being serially diluted in LB broth and counted.

According to Yang et al. [26], SGF (pH = 2.5) was prepared using the following ingredients: 8.3 g proteose peptone (Solarbio, Beijing, China), 0.6 g KH_2_PO_4_, 2.05 g NaCl, 0.37 g KCl, 0.11 g CaCl_2_ (Xilong Scientific Co., Ltd., Shantou, Guangdong, China), 0.05 g oxgall (Sigma Aldrich, St. Louis, MO, USA), 1 g lysozyme (Solarbio), and 13.3 mg pepsin (1:3000; Solarbio). Except for the oxgall, lysozyme, and pepsin, which were sterilized by filtration (0.25 µm pore size), all of the compounds were dissolved in deionized water and autoclaved together. The solution was adjusted to pH 2.5 with 5.0 M HCl. The prepared samples were mixed with SGF solution and incubated at 37 °C with shaking (130 rpm). The survival was observed, serially diluted at 10 min, and then plated on LB agar.

An oxidative stress tolerance test was conducted by inoculating the prepared samples with 50 mL LB broth containing H_2_O_2_ solution (Xilong Scientific Co., Ltd.) at a final concentration of 1, 5, and 10 mM, respectively. The samples were taken at 0, 15, 30, 60, and 120 min, and plated on LB agar.

An osmotic stress resistance test was performed by exposing strains to different concentrations of NaCI solutions (Xilong Scientific Co., Ltd.). In brief, the prepared samples were mixed with 50 mL LB broth involving 10%, 15%, and 20% NaCl (*w*/*v*) and inoculated at 37 °C. The survival was measured at 0, 60, and 120 min.

### 2.5. Superoxide Dismutase (SOD) Activity Assessment

The SOD activity was estimated using an SOD assay kit (Solarbio, Beijing, China). According to the manual, *C. sakazakii* WT strain and Δ*ptsH* mutant cultures were prepared in extraction solution and split with ultrasonication (placed on ice, ultrasonic power 20%, working time 3 s, interval 10 s, repeat for 30 times). Then, the samples were centrifuged (8000× *g*, 10 min, 4 °C) to remove insoluble materials and mixed with the regents that were provided. After incubating at room temperature for 30 min, the OD_560_ of each sample was detected.

The following formula was used to calculate the SOD activity (U/10^4^ cell):

Inhibition percentage (P) = [ΔA_B_ − ΔA_T_] ÷ ΔA_B_ × 100%,

SOD (U/10^4^ cell) = 0.0228 × P ÷ (1 − P) × F, (1)

ΔA_B_: the absorbance of (blank1 − blank 2) at 560 nm

ΔA_T_: the absorbance of (experimental group − control group) at 560 nm

F: Sample dilution multiple.

### 2.6. Specific Biofilm Formation (SBF) Assay

Biofilm formation assays were performed in accordance with the method that was described previously [27] with minor alterations. Overnight cultures of the WT strain and Δ*ptsH* mutant were diluted to OD_600_ of 1, and 1 mL of the suspension was added to sterile 24-well microtiter plates at 37 °C for 48 h without agitation. At the final time point, 200 μL of each sample was added to a 96-well microplate and the OD_630_ of cell growth were measured. After discarding the suspension, the wells were rinsed with distilled water. The wells were air dried for 30 min before being stained with 1 mL of 1% crystal violet (*wt/vol*) (Tianjin Kermel Chemical Regent Co., Ltd., Tianjin, China) at room temperature for 20 min. The dyes that were absorbed to the biofilm were solubilized in 1 mL of 33% (*vol/vol*) glacial acetic acid and incubated at room temperature for 20 min. Subsequently, the OD_570_ of each well was detected. The SBF index was calculated by correcting the OD_570_ with the OD_630_. The ability of biofilm formation was classified into two categories by SBF values: strong biofilm producers (SBF index > 1) and weak biofilm producers (SBF index < 1) [28].

### 2.7. Adhesion to HT-29 Cells

The human colonic cell line HT-29 was obtained from the Fourth Military Medical University (Xian, China) and prepared as described in a previous study [29]. The impact of *ptsH* on *C. sakazakii* adhesion to the cell surface was performed by using HT-29 cells according to a previous study [30]. Trypsin-treated cells (10^5^ cells per well) were seeded in 24 well tissue culture plates and grown in Dulbecco’s modified Eagle medium with supplements for 24 h. Both strains were centrifuged and resuspended in cell culture medium without double antibiotics. Following phosphate-buffered saline (PBS) rinsing, the HT-29 cells were added to 10^8^ CFU of WT strain or Δ*ptsH* mutant. The plates were incubated at 37 °C in a humidified, 5% CO_2_ incubator. After 2 h of incubation, the infected monolayer cells were rinsed three times in PBS and then lysed with 0.1% Triton X-100 (Amresco, Solon, OH, USA). The population of viable adherent cells was counted by serial dilution.

### 2.8. RNA-Seq Analysis

In order to determine the impact of *ptsH* gene in *C. sakazakii* BAA-894, transcriptomic analysis was performed according to previous studies [21,31]. RNA was purified respectively from the WT strain (*n* = 3) and Δ*ptsH* mutant (*n* = 3), in which both strains had been cultured earlier in LB broth with proper antibiotics and grown to an OD_600_ of 1.0. Total bacterial RNA was extracted and the RNA concentration was assessed using the nucleic acid spectrophotometer (Nano-200; Aosheng Instrument Co., Hangzhou, China). The complemental DNA (cDNA) libraries were constructed and sequenced by Novogene Technology Co., Ltd. (Beijing, China) on the Illumina sequencing platform (HiSeq™ 2500). To define a significant difference in gene expression levels, the absolute value of the log_2_ ratio ≥ 1 and *p* value < 0.05 were used as threshold values. The Kyoto Encyclopedia of Genes and Genomes (KEGG) pathway database was used to annotate all differentially expressed genes [32]. The clustered heatmaps was plotted using the “pheatmap” package (version 1.0.12) of the R software (version 4.1.1, Vienna, Austria).

### 2.9. Quantitative Real-Time PCR (qRT-PCR) Analysis

To validate the sequencing results with qRT-PCR, we selected 9 genes with differential expression between the WT and Δ*ptsH* groups (*p* < 0.05). In addition, we selected *rpoS* (RNA polymerase, sigma 38 factor), *sodA* (superoxide dismutase), *cpxR* (envelope stress response regulator transcription factor CpxR), *cpxA* (envelope stress sensor histidine kinase CpxA), and *osmY* (molecular chaperone OsmY) genes to clarify the potential mechanisms of *ptsH* that is involved in oxidative and osmotic stress regulation.

Total RNA of WT strain and Δ*ptsH* mutant was extracted from NA that was supplemented LB broth using a RNAprep Pure Bacteria Kit (Tiangen, Beijing, China) in accordance with the manufacturer’s instructions. Following the determination of RNA concentrations, cDNA was synthesized using a PrimeScript RT Reagent Kit (Takara, Kyoto, Japan). The primers that were used for qRT-PCR are listed in Table 3. The qRT-PCR reactions were carried out in a 25 μL reaction volume using SYBR Premix Ex Taq II (Takara). The cycling conditions consisted of 95 °C for 30 s, 40 cycles of 95 °C for 5 s, and 60 °C for 30 s, followed by dissociation steps of 95 °C for 15 s and 60 °C for 30 s. The ESA_04030 gene was used to normalize the gene expression levels. The samples were amplified on an IQ5 system (Bio-Rad), and the gene expression levels of all the samples were calculated according to the 2^−ΔΔCt^ method [33].

### 2.10. Statistical Analysis

All of the experiments were carried out in triplicate, and each biological replicate included three technical replicates. Statistical analyses were performed using SPSS software (version 19.0; SPSS, Inc., Chicago, IL, USA). The data were displayed as the mean values ± standard deviation. Differences are considered significant and extremely significant at *p* < 0.05 and *p* < 0.01, respectively.

## 3. Results

### 3.1. Growth Curve

**Figure 1:**
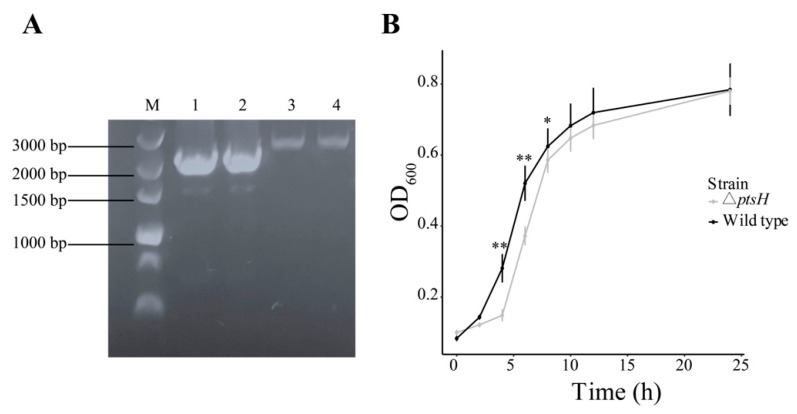
Identification of *ptsH* deletion mutant using PCR (**A**). M: DNA Ladder 5000; Lane 1, 2: Δ*ptsH* mutant; Lane 3, 4: WT strain. Growth curve of the WT strain and Δ*ptsH* mutant (**B**). Each value represents the average of three independent measurements. The bars represent the standard deviation (*n* = 3). ** *p* < 0.01, * *p* < 0.05 compared to the WT strain.

The Δ*ptsH* mutant was confirmed by PCR with the primers UP-F/DOWN-R (Figure 1A). Based on the OD_600_ values, the Δ*ptsH* strain exhibited a significant reduction in the optical density during the logarithmic phase (4–8 h) (*p* < 0.05) (Figure 1B). However, the growth resumed after 10 h (early in the stationary phase) and genetic deletion of the *ptsH* gene did not significantly influence the growth of *C. sakazakii*. 

### 3.2. The ΔptsH Mutant Decreased Tolerance to Heat and SGF

**Figure 2:**
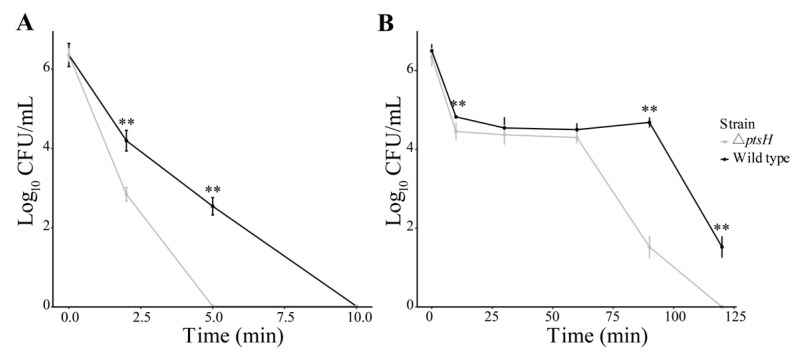
Heat resistance of WT strain and Δ*ptsH* mutant at 65 °C (**A**) and 55 °C (**B**). Bars represent the standard deviation (*n* = 6). ** *p* < 0.01 compared to the WT strain.

The ability of the WT strain and Δ*ptsH* mutant to resist heat was tested at 65 °C and 55 °C (Figure 2). When exposed at 65 °C, the WT strain dropped below the detection limit (<10 CFU/mL) within 10 min, whereas the Δ*ptsH* mutant was sharply eliminated in 5 min (Figure 2A). When exposed to 55 °C for 60 min (with the exception of time point = 10 min), the WT strain and Δ*ptsH* mutant showed no difference. However, the Δ*ptsH* mutant showed considerable susceptibility to 55 °C after 60 min and declined below the detectable level (<10 CFU/mL) at 120 min, while the WT strain remained at 1.50 log_10_ CFU/mL (Figure 2B).

**Figure 3:**
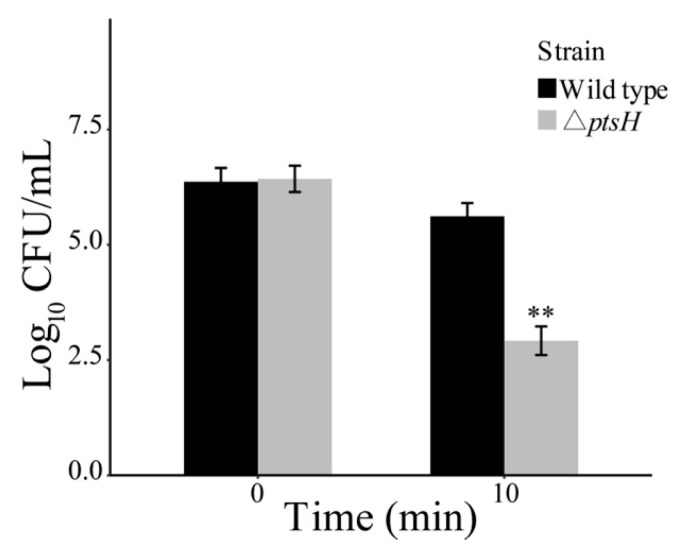
SGF tolerance of the WT strain and the Δ*ptsH* mutant. ** *p* < 0.01 compared to the WT strain.

In order to elucidate the resistance to SGF, the WT strain and Δ*ptsH* mutant were exposed to an acidic condition, a vitro model mimicking the human digestive system. The deletion of *ptsH* significantly reduced the population (2.70 log_10_ CFU/mL) of *C. sakzakii* during SGF treatment at 10 min (*p* < 0.01) (Figure 3). Thus, our findings indicate that the *ptsH* is responsible for the resistance to heat and SGF.

### 3.3. The ΔptsH Mutant Enhanced Tolerance to Osmotic Stress

**Figure 4:**
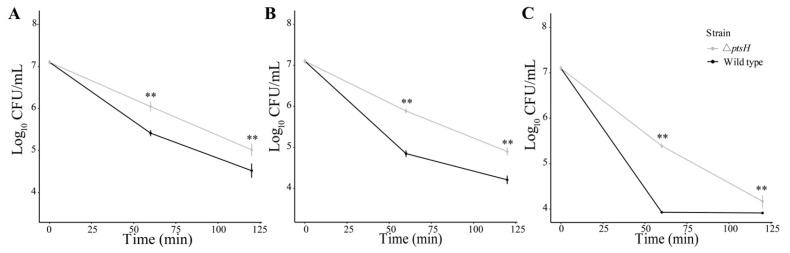
The survival of the WT strain and Δ*ptsH* mutant under 10% NaCl (**A**), 15% NaCl (**B**), and 20% NaCl (**C**). ** *p* < 0.01 compared to the WT strain.

The viability of the Δ*ptsH* mutant under high osmotic stress was investigated by different concentrations of NaCl. The WT strain has higher sensitivity to 10%, 15%, and 20% NaCl compared with the Δ*ptsH* mutant during the 120 min (Figure 4A–C). The adaption of the Δ*ptsH* mutant was enhanced by the rising osmotic pressure. The populations of the *ptsH* mutant were, respectively, 0.63, 1.04, and 1.46 log_10_ CFU/mL greater than those of the WT strain as the concentration of NaCl increased (at the 60 min time point).

### 3.4. The ΔptsH Mutant Enhanced Tolerance to Oxidative Stress

**Figure 5:**
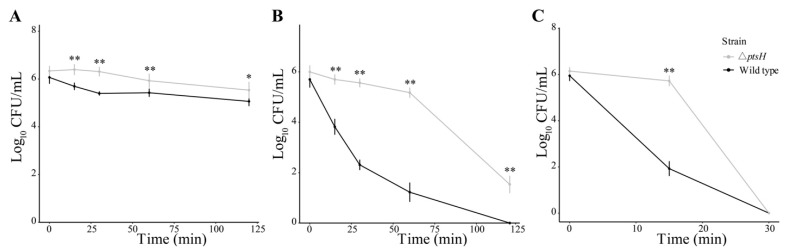
The viability of the WT strain and Δ*ptsH* mutant under 1 mM H_2_O_2_ (**A**), 5 mM H_2_O_2_ (**B**), and 10 mM H_2_O_2_ (**C**). ** *p* < 0.01, * *p* < 0.05 compared to the WT strain.

H_2_O_2_ exposure was used to compare the oxidative tolerance in the Δ*ptsH* mutant and WT strain. The Δ*ptsH* mutant showed stronger survival ability than the WT strain when treated with 1 mM, 5 mM, and 10 mM H_2_O_2_ (Figure 5A–C). For 1 mM H_2_O_2_, the distance between the Δ*ptsH* mutant and WT strain was highest (0.91 log_10_ CFU/mL) at 30 min. The population of the WT strain was significantly less than that of the Δ*ptsH* mutant during the whole treatment (*p* < 0.05) (Figure 5A). When treated with 5 mM H_2_O_2_, the WT strain was eliminated below the detectable level (<10 CFU/mL) in 120 min while the Δ*ptsH* mutant remained at 1.53 log_10_ CFU/mL (Figure 5B). When treated with 10 mM H_2_O_2_ for 15 min, the population of the Δ*ptsH* mutant remained at 5.72 log_10_ CFU/mL, while the WT decreased to 1.93 log_10_ CFU/mL (Figure 5C). 

### 3.5. The Deletion of ptsH Increased SOD Activity and Upregulated Genes Related to Oxidative and Osmotic Stress

**Figure 6:**
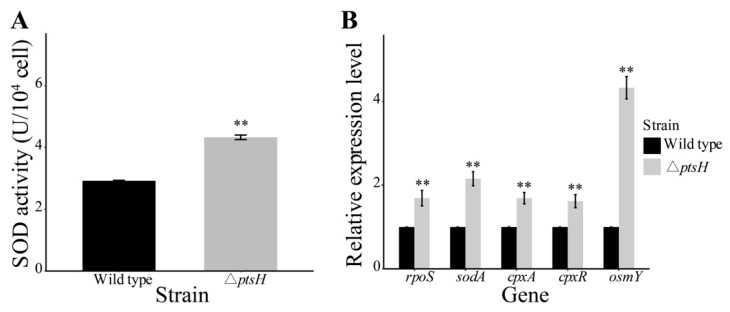
SOD activity (**A**), transcription levels of genes that were related with oxidative and osmotic stress (**B**) in the WT strain and Δ*ptsH* mutant. ** *p* < 0.01 compared to the WT strain.

SOD is a critical antioxidant enzyme that contributes to the elimination of superoxide radicals and protects against oxidative stresses [34]. In the current study, the SOD activity was enhanced (1.52-fold) in the Δ*ptsH* mutant (*p* < 0.01) (Figure 6A).

To further elaborate on the role of *ptsH* in oxidative and osmotic stress, we ran qRT-PCR of genes that are associated with these stresses in the Δ*ptsH* mutant and WT strain. The loss of *ptsH* significantly enhanced the expression of *rpoS* (1.69-fold), *sodA* (2.15-fold), *cpxR* (1.62-fold), *cpxA* (1.69-fold), and *osmY* (4.33-fold) genes in *C. sakazakii* (*p* < 0.01) (Figure 6B).

### 3.6. The ΔptsH Mutant Attenuated the SBF Index and Adhesion to HT-29 Cells of C. sakazakii

**Figure 7:**
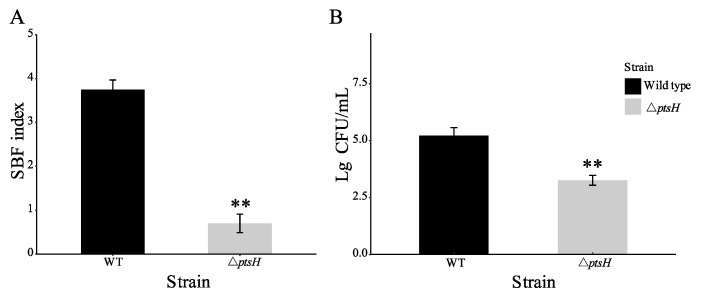
Effect of *ptsH* on biofilm formation ability (**A**) and adhesion to HT-29 cells (**B**) in *C. sakazakii* BAA-894. ** *p* < 0.01 compared to the WT strain.

To examine whether *ptsH* contributes to the virulence of *C. sakazakii*, biofilm formation and adhesion to HT-29 cells were tested. After 48 h incubation, the WT strain developed a robust biofilm (SBF index = 3.75), whereas the Δ*ptsH* mutant showed a significant deficiency (SBF index = 0.60) in biofilm formation (*p* < 0.01) (Figure 7A). After co-incubation with HT-29 cells for 2 h, the adhesion of the WT strain and Δ*ptsH* mutant were detected (Figure 7B). In the absence of *ptsH*, *C. sakazakii* adhesion was significantly decreased (relative 1.96 log_10_ CFU/mL) (*p* < 0.01) (Figure 7B).

### 3.7. Overall Transcriptome Comparison of WT Strain and ΔptsH Mutant 

**Figure 8:**
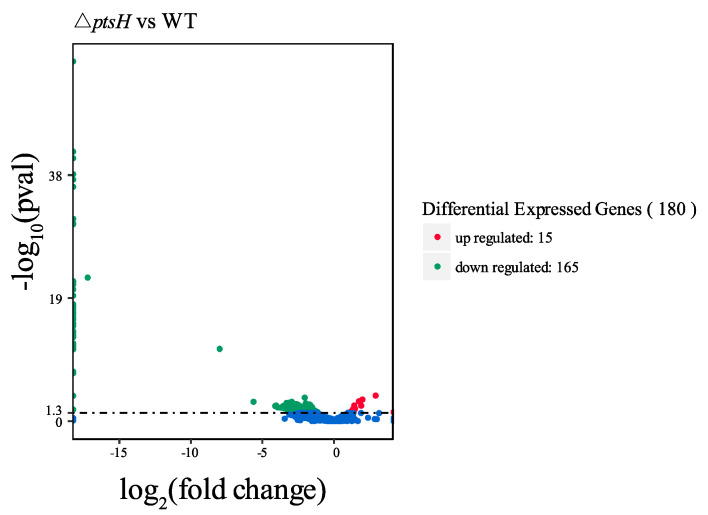
Volcano map of differentially expressed genes between the WT strain and the Δ*ptsH* mutant. Red dots represent the upregulated genes, green dots represent the downregulated genes, and blue dots represent the genes with no significant differential expression.

To expand our understanding of the genetic variation that was regulated by *ptsH*, we performed RNA-seq. The volcano plot revealed that 180 genes in the *ptsH* mutant were differentially expressed, with 15 that were upregulated and 165 downregulated (|log_2_ (fold change)| > 1, *p* < 0.05) (Figure 8).

**Figure 9:**
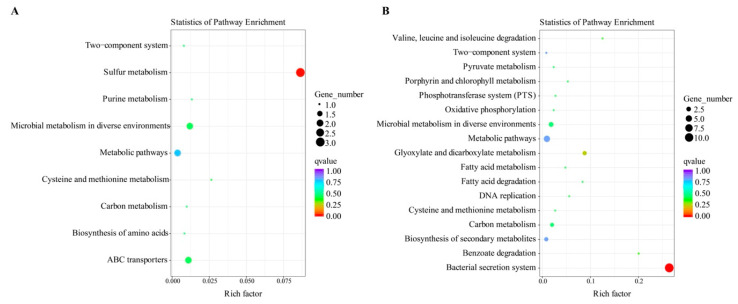
KEGG pathway enrichment analysis of the upregulated (**A**) and downregulated (**B**) mRNAs.

There were 26 KEGG pathways that were enriched in total, and the upregulated (*n* = 9) and downregulated (*n* = 17) pathways are summarized (Figure 9A, B). The primary pathway that was downregulated in the Δ*ptsH* mutant was the bacterial secretion system, whereas the sulfur metabolism pathway was markedly increased (*P*_adj_ < 0.01). 

### 3.8. Analysis of Significant Differential Pathways

**Figure 10:**
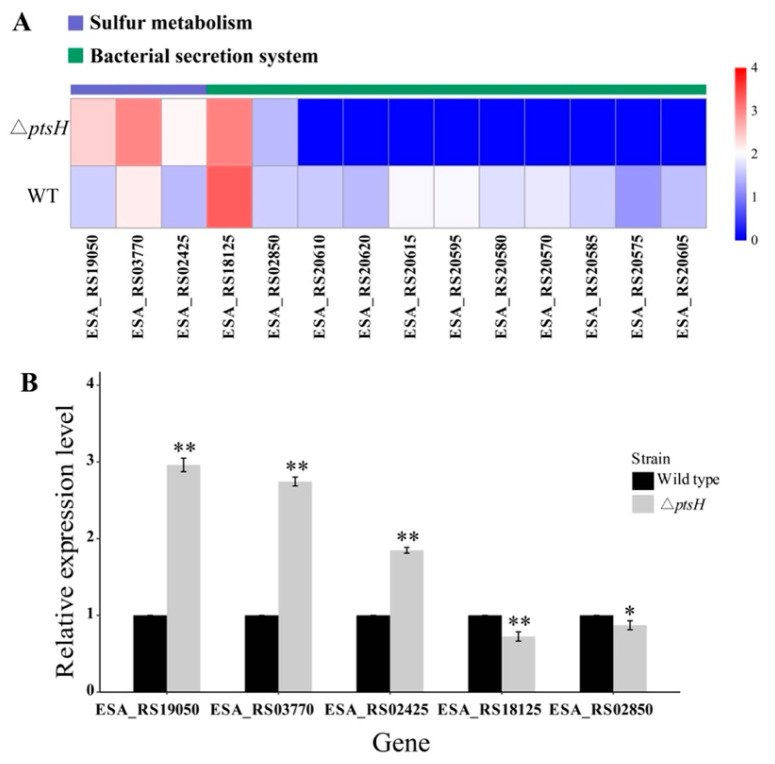
Heatmap of differentially expressed genes that were assigned to sulfur metabolism and bacterial secretion systems in the WT strain and Δ*ptsH* mutant (**A**). The color of the bar corresponds to different ranges of log_10_(FPKM+0.1). FPKM: fragments per kilobase of transcript sequence per millions base pairs that were sequenced. Transcription levels of genes that were related to sulfur metabolism and bacterial secretion systems in the WT strain and Δ*ptsH* mutant (**B**). ** *p* < 0.01, * *p* < 0.05 compared to the WT strain.

For upregulated pathways, three genes were enriched in sulfur metabolism, including genes that were associated with sulfate ABC transporter substrate-binding protein (ESA_RS19050), cysteine synthase A (ESA_RS03770), and adenylyl-sulfate kinase (ESA_RS02425) (Figure 10A). The qRT-PCR data revealed that the relative expressions of the genes that were mentioned above were increased by 1.96-, 1.74-, and 0.85-fold in the Δ*ptsH* mutant, respectively (Figure 10B).

Among the downregulated pathways, 11 genes were assigned to the bacterial secretion system pathway, and most of the genes were related to Type VI (ESA_RS18125, ESA_RS20570, ESA_RS20615), Type IV (ESA_RS20585, ESA_RS20605), and Type I (ESA_RS02850) secretion systems (Figure 10A). According to qRT-PCR results, the relative expressions of ESA_RS18125 (encoding Hcp1 family Type VI secretion system effector) and ESA_RS02850 (encoding Type I secretion protein) were reduced by 0.38- and 0.13-fold in the Δ*ptsH* mutant, respectively (Figure 10B).

## 4. Discussion

Since *C. sakazakii* isolates have been reported to exhibit a strong tolerance to numerous stressful conditions and cause PIF contamination and neonatal infections [5,35], it is crucial to have a better knowledge of the mechanisms underlying their stress response and virulence. PTS is not only important for carbohydrate metabolism but also regulates the adaptive response of bacteria to harsh stress and virulence [12,15]. In the current study, the functional characterization of *ptsH* in *C. sakazakii* BAA-894 is examined for the first time. 

In this study, the growth curve revealed that the Δ*ptsH* mutant has lower cell density during the logarithmic phase compared with the WT strain. Quorum sensing (QS) is a bacterial process that regulates relative population densities [36]. The absence of HPr was shown to increase the LsrK kinase activity, which resulted in a decrease in the extracellular autoinducers-2 (small signaling molecules mediating QS) and delayed cell growth [12]. We hypothesize that the repression of the QS signal that was caused by the deficiency of *ptsH* is a potential explanation for the slow growth rate in the early phase.

However, bacterial growth was recovered after 10 h, and the cell population of the Δ*ptsH* mutant was almost identical to that of the WT strain. This result demonstrated that *C. sakazakii* can grow independent of *ptsH* modulation. Similar patterns in the *ptsH* mutant of other bacteria have been discovered in several studies. The *ptsH* deletion mutant in *Staphylococcus aureus* reduced the growth rate in the exponential (1–3 h) and the transition phase (4–6 h) relative to the WT strain, while the growth rates were comparable for both strains after 12 h of cultivation [37]. The ultimate biomass of the *ptsH* deletion mutant (in *E. coli*) was 30.51% more than that of the WT strain, which was expected given that the inactivation of HPr requires recruiting a non-PTS transport mechanism such as the galactose permease system [38]. In addition, bacteria will develop redundant metabolic systems, such as nitrogen and sulfur metabolism, to maintain development in a nutrient-restricted environment. It has been demonstrated that 49 sulfur metabolism genes were shared by *C. sakazakii* ATCC BAA-894 and *C. sakazakii* SP291 [39]. According to our RNA-seq data, the sulfur mechanism pathway was considerably elevated in the Δ*ptsH* mutant (*p* < 0.01, *P*_adj_ < 0.01). The expressions of three of the genes that were involved in this pathway were highly elevated in the Δ*ptsH* mutant, according to the results of qRT-PCR (*p* < 0.01).

Heat and acid resistance are important characteristics of *C. sakazakii* for its survival in harsh environments, including manufacturing facilities and GIT [40,41]. According to Huang et al. [42], *Lactobacillus plantarum* exhibits higher transcription of the *ptsH* gene under acid stress than under control conditions. Similar to this, the loss of the *ptsH* gene in *C. sakazakii* BAA-894 led to a high sensitivity to high temperatures (65 °C and 55 °C) and acid stress (pH = 2.5).

The bacterial Type VI protein secretion system (T6SS) is crucial for cell survival following exposure to a variety of environmental stressors [43]. According to an earlier study, T6SS4 contributes to acid survival by maintaining a steady-state intracellular pH in *Yersinia pseudotuberculosis* [44]. The deletion of T6SS reduced the tolerance of *Campylobacter jejuni* to deoxycholic acid (DCA) by increasing the intracellular influx of DCA [45]. In this study, the RNA-seq results revealed that the bacterial secretion system, which includes genes that are associated with T6SS, is the most significantly downregulated pathway in the Δ*ptsH* mutant (*p* < 0.01, *P*_adj_ < 0.01). Similarly, almost all of the T6SS genes in *Edwardsiella piscicida* had considerably decreased levels of transcription in Δ*ptsH* mutant than those in WT [18]. Hcp (hemolysin-coregulated protein) is regarded as a pivotal component of the functional T6SS [46]. According to the qRT-PCR results, the relative expression of ESA_RS18125, which encodes Hcp1 family T6SS effector, was reduced in Δ*ptsH* mutant compared with the WT strain. We propose that the loss of the *ptsH* gene downregulates the expression of T6SS possibly by suppressing the Hcp protein. Additionally, the XRE family has been identified as a critical regulator for acid adaption in *Lactobacillus acidophilus* [47], and has been linked to *Arthrospira platensis* survival under high temperature stress [48]. Our RNA-seq data revealed that the deletion of the *ptsH* completely eliminates the expression of the genes encoding xenobiotic-responsive element (XRE) family transcriptional regulator. 

Osmotic stress is typically encountered by *C. sakazakii* during food production (especially in PIF), whereas oxidative stress is also common in the food processing environment and human immune cells [49,50]. In the current study, it was discovered that the Δ*ptsH* mutant had a great capacity of survival under oxidative and osmotic stressors. Similar to this, *Neisseria meningitidis* showed an upregulation of numerous proteins that are implicated in redox reactions when *ptsH* was absent [15].

SOD is a critical antioxidant enzyme that is found in numerous bacteria that protects cells from oxidative stress and the removal of superoxide radicals [34]. A prior study found that the deficiency of the *ptsH* gene inhibited the transcription of *sodA2* gene in a *Bacillus cereus* strain [14]. In contrast to the current findings, the Δ*ptsH* mutant had increased the SOD activity and the expression of the *sodA* gene by 1.52-fold and 2.15-fold, respectively. It implies that the ability of *ptsH* to withstand oxidative conditions varies depending on the species. To further elucidate the potential mechanism of strong oxidative and osmotic resistance in the Δ*ptsH* mutant, we investigated the relative expression of *rpoS*, *cpxR*, *cpxA*, and *osmY*.

In Gram-negative bacteria, the general stress response of stationary phase cells is modulated by the alternative sigma factor RpoS [51]. It was reported that RpoS appears to be the main signal that regulates the response of bacteria to high oxidative and osmotic pressure conditions [52,53]. In this study, the *rpoS* gene was 1.69-fold enhanced in the Δ*ptsH* mutant (*p* < 0.01). It can be concluded that increasing *rpoS* expression is another adaptive strategy in high-osmolality environments for *C. sakazakii* BAA-894 Δ*ptsH* mutant. The Cpx system, known as an envelope stress response regulator, plays an important role in sensing changes in protein folding and improving the cell envelope integrity [54]. Salt sensitivity was observed in mutants whose genes encoding CpxR and CpxA were disrupted [55]. In addition, OsmY is an osmotically-inducible protein, and has been shown to be dependent upon *rpoS* for expression by osmotic stimuli [56]. Our qRT-PCR data revealed the transcriptional levels of *cpxR*, *cpxA,* and *osmY* were dramatically increased in the Δ*ptsH* mutant (*p* < 0.01). According to these findings, the high tolerance of the Δ*ptsH* mutant to oxidative and osmotic conditions may be due to the increased expression of *rpoS*, Cpx system, and *osmY*. Previous research found that certain metabolic pathways, such as amino acid metabolism and energy metabolism, respond to oxidative and osmotic stressors [17,57]. Cysteine and methionine, as the most representative sulfur-containing amino acids, can restore oxygen tolerance [58], while purines act as energy carrier molecules [59]. In this study, our RNA-seq and qRT-PCR results revealed that the gene ESA_RS03770 (cysteine synthase A), which is involved in cysteine and methionine metabolism, amino acids biosynthesis, and the purine metabolism pathway, was considerably upregulated in the Δ*ptsH* mutant (*p* < 0.01). 

The adherence of pathogens to the surface of cells is the first step of the colonization of tissues [60]. A greater capacity for biofilm formation by bacteria suggests a greater capacity for adherence [61]. This study used human colonic cell line HT-29 to verify the influence of the *ptsH* gene on the pathogen-host interaction. The results demonstrated that the deletion of the *ptsH* gene dramatically reduced the ability of bacteria to produce biofilm and to adhere, indicating the significant impact of the *ptsH* gene on these two phenotypes. Furthermore, several studies have shown that the defective *ptsH* gene inhibits biofilm formation and infection in other bacteria [37,62].

Previous research identified pyruvate as a pivotal substance in metabolic pathways during biofilm formation by modulating cAMP phosphodiesterase activity [63]. According to our RNA-seq results, *ptsH* deficiency in *C. sakazakii* reduced the transcription of the ESA_RS00240 gene (involved in the pyruvate metabolism pathway; -2.54-fold; *p* < 0.05). Many microbes require adhesin, a cell surface-associated protein, to initiate surface contact and biofilm formation [64]. It has been reported that T1SS (localized on the cell surface) secretes a variety of adhesins to facilitate contacts between the cells and surfaces [65]. Besides the stress response, T6SS is also involved in biofilm formation and interrelations with host epithelial cells in several bacterial pathogens [66,67]. Our RNA-seq findings showed ESA_RS13590 (encoding Type I toxin-antitoxin system hok family toxin), ESA_RS02850 (encoding Type I secretion protein), and ESA_RS18125 (encoding Hcp1 family Type VI secretion system effector) were downregulated in the Δ*ptsH* mutant by 1.57-, 1.42-, and 1.82-fold, respectively (*p* < 0.05). Further research is required to determine how *ptsH* regulates pyruvate metabolism, bacterial secretion systems, and adhesins.

There are certain limitations in this study. Firstly, we studied the transcriptional changes in WT and mutants that were grown only in optimal conditions. Future research comparing the transcriptional differences between the WT strain and Δ*ptsH* mutant that are grown in different stressed conditions will better clarify the genetic basis for *ptsH*’s contribution to the response to each specific stress. Secondly, we failed to complement the *ptsH* gene in the mutant after several attempts using pBAD expression plasmid. We will test more plasmids for complementation in our future studies examining the additional function of *ptsH* or other genes in *C. sakazakii*. We hope that the current findings using the deletion mutant [31,68] could still contribute to our understanding the role of *ptsH* in *C. sakazakii*.

In this study, we examined the impact of *ptsH* deletion on growth, stress response, and virulence in *C. sakazakii* BAA-894. The *ptsH* gene impaired the growth rate of *C. sakazakii* only in the early log phase. Moreover, the heat stress tolerance, survivability in SGF, biofilm formation, and adhesion to HT-29 cells of the Δ*ptsH* mutant were significantly decreased, while the SOD activity and the resistance to oxidative and osmotic stress were enhanced. *SodA*, *rpoS*, *cpxA*/*R*, *osmY,* and genes that are related to the sulfur metabolism pathway were upregulated in the Δ*ptsH* mutant, while the genes that were assigned to bacterial secretion systems, such as T1/T4/T6SS, were downregulated. These findings enrich our knowledge of the role of *ptsH* in diverse stress responses and virulence in *C. sakazakii*, and it contributes to our understanding of genetic determinants of stress resistance and pathogenicity of this important foodborne pathogen.

## Figures and Tables

**Table 1:** Bacterial strains and derivative plasmids that were used in this study.

Strains and Plasmids	Relevant Characteristics	Sources
*E. coli* S17 *λpir*	*Tpr Smr recA thi pro rK- mK- RP4:2-Tc:MuKm Tn7 λ pir (thi pro hsdRhsdM + recA RP4-2-Tc:: Mu-Km-Tn7)*	[23]
*Cronobacter sakazakii*		
BAA-894	WT, NA-induced mutant of *C. sakazakii* ATCC BAA-894, NA^r^	Laboratory collection
Δ*ptsH*	*ptsH* deletion mutant of BAA-894, NA^r^	This study
Plasmid		
pDM4	Suicide vector, *mob*RK2, *ori*R6K, *pir*, *sacB*, Cm^r^	[24]
pDM4::UP+DOWN	Construct used for in-frame deletion of *ptsH*, Cm^r^	This study

Na^r^ and Cm^r^ represent resistance to nalidixic acid and chloramphenicol, respectively.

**Table 2:** Primers that were used in this study.

Primer	Sequence (5′-3′)
UP-F	ACATGCATGCGAAAGCGGAAGAGATT
UP-R	GCTGGAACATTGTATTTCCCC
DOWN-F	GGGGAAATACAATGTTCCAGCGAACTCGAGTAAGTTCC
DOWN-R	ACGCGTCGACCTCTTCTTCGGTT

**Table 3:** Primers that were used for RT-qPCR analysis in this study.

Primer	Sequence (5′-3′)
ESA_04030	F, CCAGGGCTACACACGTGCTA
	R, TCTCGCGAGGTCGCTTCT
*osmY*	F, CGCGAAGGAACGATGTCAT
	R, CCACCACCAGCGAAATCAA
*rpoS*	F, CTGGTGGATTCGTCAGACCAT
	R, GCGAATCGTACGGGTTTGG
*cpxR*	F, TTGAGCTGGGCGCGGATGAT
	R, TGCCGAGCACTTCCTGGCTT
*cpxA*	F, ACCGCCCGCATCTTCGCCATTT
	R, AACAACCGCCGCCACCACATCA
*sodA*	F, CGAATCTGCCGGTTGAAGA
	R, CTTGTCCGCCGGAACCT
ESA_RS19050	F, TATCCTCGCCGAGCCGACTGTT
	R, CAGCCGCCGAACACGTCATCAA
ESA_RS03770	F, TGGAGTCACGTAACCCAAGC
	R, CGACGGTAATCAGCTCGGTT
ESA_RS02425	F, CTTTCGGGCTCCGGAAAATC
	R, TCAGGATTTGATGATATCTTCGCG
ESA_RS18125	F, GGCTGGACCGACATCACCTCTT
	R, CGCTGGAGCAGTGCTTCAGGAT
ESA_RS02850	F, GACACGCTTCTGGATGAGGT
	R, TCCCTTAACGCCTCTTTCACC

F, forward primer; R, reverse primer.

## Data Availability

The data presented in this study are available on request from the corresponding author.
